# ZIF‑8 Microswimmers Self-Fast Dynamic Destruction in Microliter Volumes of Cerebrospinal Fluid Samples: Toward a Selective Assessment of Amyloidosis

**DOI:** 10.1021/acs.analchem.5c03467

**Published:** 2025-08-15

**Authors:** Javier Bujalance-Fernández, Eva Carro, Desiree Antequera, Beatriz Jurado-Sánchez, Alberto Escarpa

**Affiliations:** † Department of Analytical Chemistry, Physical Chemistry and Chemical Engineering, 98711 Universidad de Alcala, E-28802 Alcala de Henares, Madrid, Spain; ‡ Chronic Disease Programme, UFIEC, Carlos III Health Institute, E-28029 Majadahonda, Madrid, Spain; § CIBERNED, E-20031 Madrid, Spain; ∥ Chemical Research Institute “Andres M. del Rio”, Universidad de Alcalá, E-28802 Madrid, Spain

## Abstract

Herein, we describe the synthesis of magnetic zeolitic imidazole framework (ZIF-8) microswimmers for detecting and quantifying the amyloid beta (Aβ_1–42_) peptide in cerebrospinal fluid (CSF) samples, which are used as a biomarker of amyloidosis for diagnosing Alzheimer’s disease (AD). The microswimmers are prepared by external decoration of the ZIF-8 with magnetic Fe_3_O_4_ nanoparticles, followed by post internal encapsulation of quinine as fluorescent probe. The magnetic and surface properties of the microswimmers are fine-tuned to obtain tailored structures with an inner porous structure with the loaded fluorescent probe and outer magnetic engines. A macroporous structure was preferred over a microporous structure for quinine encapsulation, increasing the loading efficiency by about 50%, allowing also external decoration of the ferrite to impart the desired magnetic properties to the microswimmer. The principle for detection relies on the specific affinity of target sequence of amino acids in the Aβ_1–42_ peptide structure toward the Zn units in the ZIF-8, resulting in the self-destruction of the microswimmers and subsequent release of quinine in a concentration-dependent manner. The use of the bioreceptor-free magnetic assisted microswimmers allows for direct assessment of Aβ_1–42_ peptide in only 10 μL of CSF samples in just 10 min. Excellent analytical performance with a limit of detection of 40 pg/mL and a linear range ranging from 140 to 1200 pg/mL (r = 0.9990), covering the range in the clinical practice, is obtained. An excellent selectivity was also obtained toward the Aβ_1–42_ peptide which approaches an excellent assessment of amyloidosis in the human brain, as demonstrated by the good correlation obtained (r = 0.97) between the quantitative levels obtained in our microswimmers approach in comparison with the enzyme-linked immunosorbent assay method in diagnosed CSF samples from patients where Tau protein was also determined due to its coexistence with Aβ_1–42_ peptides. Since CSF biomarkers are currently the only clinically validated biofluid diagnostic test for AD, our approach will drastically reduce the volume required to determine Aβ levels, reducing the impact of the side effects of lumbar puncture in clinical practice. It became a novel bioreceptor-free approach to more easily, less invasively measure Aβ_1–42_ peptide in CSF, becoming a valuable tool for indirect amyloidosis prediction in brain tissues in the patient’s lifetime, opening the possibility for early treatment of the AD.

## Graphical abstract

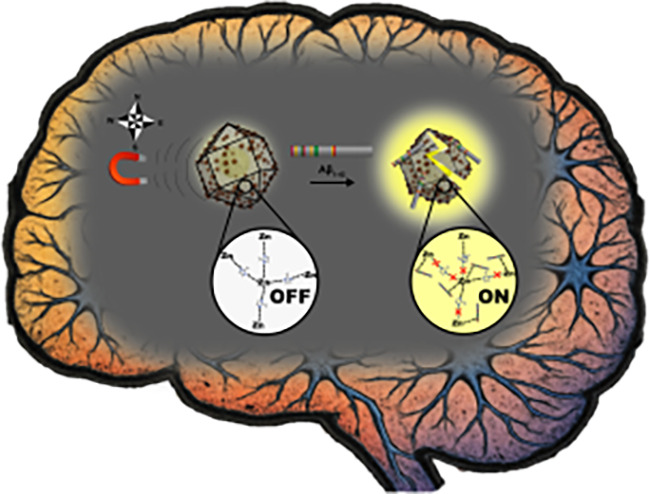

## Introduction

The design and development of vanguard bioanalytical approaches for the diagnosis and monitoring of Alzheimer’s disease (AD) is a complex matter involving low sample availability due its invasive nature (i.e., collection of cerebrospinal fluid samples, CSF), the inherent complexity of the media, and the presence of labile analytes that are difficult to detect.^1^ Among the most prevalent AD biomarkers are amyloid-β (Aβ) peptides and tau protein, which can generate plaques that irreversibly damage the brain tissue.^2,3^ The detection of Aβ peptides in CSF is crucial for early diagnosis and monitoring, accounting for the limited clinical treatments to prevent the disease. CSF biomarkers are currently the only clinically validated biofluid diagnostic test for AD.^4,5^ Although CSF biomarker testing is routinely used in the diagnosis of multiple conditions in clinical neurology, it involves the invasive procedure of lumbar puncture (LP), which is often associated with headaches. Post-LP headache is mainly due to the volume of fluid extracted (>10 mL). Therefore, techniques that can drastically reduce the volume of fluid required to determine Aβ levels are needed to reduce the impact of the side effects of lumbar puncture.

Micromotors or microswimmers are microscale devices that represent a fascinating approach in the current analytical chemistry scenario, on account of their enhanced self-propulsion abilities and controlled motion, which allow for detection in a few microliters of sample.^6–11^ Such unique features allow for dynamic interactions with the analytes holding considerable promise in microscale environments and opening new avenues for on-the-fly (bio)-sensing strategies,^12–14^ which have recently been critically discussed.^15^

Tubular catalytic micromotor-based bioassays for AD diagnosis have been explored using both electrochemical immunoassays for Aβ_1–42_ ^16^ and for the dual determination of Aβ_1–42_ and tau^17^ as well as aptassays for the Aβ_1–42_ oligomer^18^ in low volumes of hardly available clinical samples such as brain tissue, CSF, and plasma samples with excellent agreement with gold standard methods. Such methods provide low detection limits (in the pg/mL range), which along with the low volume of sample required make them suitable approaches for fast and potential early diagnosis of AD. Yet, bioreceptors are used, implying difficulties in full biofunctionalization in the external layer of the micromotors, stability, and cross-reactivity. Therefore, as alternative high-selective approaches, bioreceptor-free methods need to be explored.

Recent efforts are aimed at exploring the combination of micromotors with novel materials to enhance the biocompatibility and locomotion abilities. In this context, metal organic frameworks (MOFs) are ideal platforms for coupling with micromotor technology.^19–22^ The use of MOFs for micromotor design was first explored by the community back in 2012 when the Marangoni-based propulsion of Cu-JAST MOFs was illustrated.^23^ MOFs are also particularly attractive for the design of biosensors in AD diagnosis due to their large specific surface area, diverse functions, and density of active sites for probe encapsulation, as illustrated in recent studies, most of them targeting Aβ peptides or oligomers. Electrochemical and optical detection approaches are the most explored, using in some cases aptamers and antibodies as specific recognition probes.^1,24^ As such, it is possible to encapsulate colorimetric or electrochemical probes into ZIF-based MOFs, which are subsequently disassembled in the presence of the target Aβ peptides in a concentration-dependent manner, as a relevant example in the field of AD diagnosis. This fact has formed the basis for indirect detection of such biomarkers using ferrocene-encapsulated ZIF-8 MOFs^25^ or to monitor intracellular adenosine triphosphate detection using rhodamine B encapsulated in ZIF-90 MOFs.^26^ In this context, our group has illustrated the feasibility of catalase-propelled ZIF-8 micromotors for motion-based copper detection in CSF from patient samples, yielding promising results that allow differentiation by groups of healthy individuals (or in early stages of Alzheimer’s disease) from others in more advanced stages.^27^ However, except for the latest work reported by the authors, micromotor-MOF coupling has not been explored in AD diagnosis. In AD, Aβ is deposited in the brain in plaques with a higher plaque burden in advanced AD stages. As insoluble Aβ_1–42_ accumulates in the brain during AD progression, there is a corresponding decrease of soluble Aβ_1–42_, as measured in the CSF of patients with AD.^28^ The National Institute on Aging and the Alzheimer’s Association framework for the diagnosis of AD requires the demonstration of high insoluble Aβ_1–42_ in the brain measured by positron emission tomography (PET) or of low soluble Aβ_1–42_, measured in CSF.^29^ However, a significant number of amyloid PET-positive individuals will not develop dementia during their lifetime.^29^ In this situation, the quantification of soluble Aβ_1–42_ in the CSF correlates better with cognition offering further information about neuropsychological performance^30^ and protein production and clearance.^29^ Herein, in line with previous information and inspired by such previous work, bioreceptor-free ZIF-8 magnetic microswimmers are designed and prepared by assembling magnetic Fe_3_O_4_ nanoparticles in the outer structure of the ZIF-8, followed by inner encapsulation of the quinine probe. We will illustrate the controlled self-disassembly of the ZIF-8 quinine encapsulated microswimmers by the Aβ_1–42_ peptide in CSF samples assisted by their magnetic properties for the analysis of CSF samples from healthy individuals and diagnosed patients. Although ZIF-8 microswimmers are not as specific as antibodies or aptamers, our approach is specific enough to differentiate between the most relevant proteins in CSF, amyloid-β and Tau proteins. Hence the relevance of this study in which we provide a novel bioreceptor-free approach to more easily, less invasive, measure Aβ_1–42_ peptide in CSF, becomes a valuable tool for indirect amyloidosis prediction in brain tissues in the patient’s lifetime, opening the possibility of treatment of the disease. While most conventional biosensors rely on antibody- or aptamer-based specific recognition, these biosensors typically necessitate intricate immobilization procedures and are vulnerable to biological instability and biofouling. In contrast, this bioreceptor-free approach offers a simpler fabrication process and greater long-term stability. Furthermore, this elegant approach enables both analysis from low sample volumes and bioreceptor-free detection. This dual innovation represents a promising step toward the development of novel early diagnostic approaches for AD.

## Experimental Section

### Synthesis of ZIF-8 Microswimmers Externally Decorated with Fe_3_O_4_

A 50 mM solution of Zn (NO_3_)_2_·6 H_2_O in methanol was mixed with a solution containing 200 mM of 1-methylimidazole, 200 mM of 2-methylimidazole, and 10 μL of polystyrene (PS) nanoparticles in methanol. The mixture was covered and incubated overnight at room temperature. Any glass or magnetic Teflon used in this incubation synthesis was previously cleaned with aqua regia to avoid any external particles that could act as aggregation points. After obtaining the ZIF-8 powder, to remove the PS encapsulation, the ZIF-8 powder was resuspended in dimethylformamide (DMF), sonicated for 1 min in an ultrasonic bath under degassing conditions, and incubated for 1 h at 900 rpm at 50 °C using a hydrophilic polytetrafluoroethylene membrane to wash and isolate the ZIF-8 under vacuum conditions. The precipitate was resuspended, collected, and washed 3 times with methanol through polycarbonate membranes under vacuum conditions. Finally, the ZIF-8 powder was obtained. For ZIF-8 functionalization, poly­(sodium 4-styrenesulfonate) (PSS)-modified iron oxide nanoparticles (II, III, or III) were used. To this end, 5 mg of the iron oxide nanoparticles were mixed with 10 mL of a 0.3% PSS solution and subsequently sonicated with an ultrasonic processor for 20 min at 80% of its power with 1 s off for every 2 s on. The iron oxide functionalized nanoparticles were washed 3 times with water held by a magnetic rack. To obtain the ZIF-8@iron oxide microswimmers, the ZIF-8 powder was mixed with the appropriate volume of the iron oxide-PSS in methanol to get the desired final concentration using an ultrasonic processor for 20 min at 80% of its power with 1 s off for every 2 s on. The sonicated sample was washed 2 times with methanol held by a magnetic rack, dried, and collected with polycarbonate membranes under vacuum conditions.

### Quinine Encapsulation and Release for Aβ_1–42_ Quantification Experiments

Prior to encapsulation, the microswimmers were dried overnight at 200 °C to eliminate residual moisture in the pores. The powder microswimmers were then incubated in 1 mL of quinine hydrochloride (25 mM) dissolved in DMF per 1 mg of ZIF-8 microswimmers. Incubation was carried out in the dark. The incubation process was started with 1 min in the degassing mode of the ultrasonic bath, followed by 1 h at 50 °C and 900 rpm. The microswimmers were then washed 3 times with water using the magnetic rack for holding them and finally allowed to dry.

### Aβ_1–42_ – Microswimmers Incubation Time Optimization

Quinine-encapsulated microswimmers were resuspended at 4 mg/mL in a defined volume of ACSF fortified with 1000 pg/mL. This suspension of microswimmers in ACSF with Aβ_42_ was incubated under magnetic conditions (550 rpm) at 37 °C in the dark for 1, 10, 30, 60, and 120 min. After this time, microswimmers release quinine due to structural degradation; therefore, iron oxide nanoparticles also start to detach from ZIF-8. To avoid interference during the measurement, the samples are filtered with nylon filters to keep only the supernatant part before reading. One μL of the supernatant was dropped onto a glass slide and mixed with 1 μL of 0.1 M H_2_SO_4_ (to improve the analytical signal). This drop was photographed with an exposure time of 200 ms using a 20× objective and a DAPI-5060C filter cube, and the sample was excited at 385 nm with an LED light source at 100%. The results were obtained from 3 photographs using the NIS program to measure the total fluorescence of the image.

### Aβ_1–42_ – Microswimmers Quinine Release Calibration Plot

Microswimmers were resuspended in a defined volume of ACSF fortified with 0, 200, 400, 600, 800, 1000, and 1200 pg/mL of Aβ_1–42_. This suspension of microswimmers in ACSF with Aβ_1–42_ was incubated under magnetic conditions (550 rpm) at 37 °C in the dark for 10 min. After this time, microswimmers release quinine due to structural degradation; therefore, iron oxide nanoparticles also start to detach from ZIF-8. Ten μL of standard solutions were filtered with nylon filters to retain the micromotors, and then repetitive individual supernatant aliquots of 1 μL each were measured by triplicate.

### Aβ_1–42_ – Microswimmers Interfering Proteins

Microswimmers were resuspended in a defined volume of ACSF fortified with 1000 pg/mL of Aβ_1–42_, Aβ_25–35_ fragment, Aβ oligomer, and Tau protein or 600 pg/mL of a mixture of Aβ_1–42_ + Aβ oligomer or Aβ_1–42_ + tau protein. This suspension of microswimmers in ACSF was incubated under magnetic conditions (550 rpm) at 37 °C in the dark for 10 min. After this time, the microswimmers release quinine due to structural degradation, and therefore the iron oxide nanoparticles also start to detach from ZIF-8. Then measurements were carried out as indicated for calibration studies.

### AD Sample Collection and Analysis

In this study, we included three groups of donors: MCI patients (n = 4), AD patients (n = 6), and health controls (n = 3) recruited from Neurology service of the University Hospital 12 of October (Madrid, Spain) (Table S1). Diagnoses were based on detailed clinical assessments, neuropsychological studies, neuroimaging, electromyography, and cerebrospinal fluid (CSF) Aβ_1–42_ and tau levels, according to the National Institute on Neurological Disorders and Stroke and the Alzheimer’s Disease and Related Disorders Association guidelines.^31^ Disease severity was evaluated using the Mini–Mental State Examination (MMSE) scores. Functional impairment was measured via the Clinical Dementia Rating (CDR) score. CSF samples were obtained by lumbar puncture on informed patients’ consent according to the Declaration of Helsinki, and approval was obtained from the Research Ethic Committee (CEIm2018/459). All samples were spun at 3000 rpm at 4C for 10 min, aliquoted in small volumes, and stored in low-bind polypropylene tubes at −80 °C.

Levels of endogenous Aβ_1–42_ in CSF samples using ZIF-8 microswimmers were determined using 10 μL of CSF which were filtered with nylon filters to retain them, and then repetitive individual supernatant aliquots of 1 μL each were measured in triplicate to improve analysis reliability. Levels of endogenous Aβ_1–42_ and total tau (t-tau) in CSF samples were also determined using the Aβ_42_ human ELISA Innotest kit (Innogenetics) and tau human ELISA Innotest kit (Innogenetics), respectively.

### Statistical Analysis

The statistical significance between the studied materials and the corresponding control experiments was evaluated by performing *t* tests (α = 0.05) with the Bonferroni correction. The statistical significance between microporous and macroporous ZIF-8 microswimmers was evaluated by performing a two-tailed *t* test with Welch correction (*p* < 0.05). All the statistical analyses were performed using the OriginPro software for data manipulation and analysis.

### Safety Statement

No unexpected or unusually high safety hazards were encountered.

## Results and Discussion

### ZIF-8-Based Microswimmers Design and Detection Principle

Figure 1A illustrates the principle of detection of the Aβ_1–42_ peptide with the ZIF-8-based magnetic microswimmers. A schematic of the structure of the peptide is also depicted and consists of an N-terminal hydrophilic part (positions 1 to 16) and a C-terminal hydrophobic part (positions 17 to 42). Aβ_1–42_ is able to bind Zn ions via the N-terminal amine, the side chains of the carboxylic acid residues at positions 1 (Asp), 3 (Glu), 7 (Asp), and 11 (Glu), and the side chains of the three His residues at positions 6, 13, and 14.^32^ Glu and Asp in positions 22 and 23 of the C-terminal part can also bind to Zn^2+^.^32^ The Zn^2+^ ions in the ZIF-8 bind to the Aβ_1–42_ peptide, particularly in the amino acids His, Glu, and Asp, due to their strongest affinity, resulting in the disassembly of the MOF units and consequently the release of the encapsulated quinine in a concentration-dependent manner.^25^ This detection principle has not been previously explored using micromotor-based *on-the-fly* bioassays.

**Figure 1:**
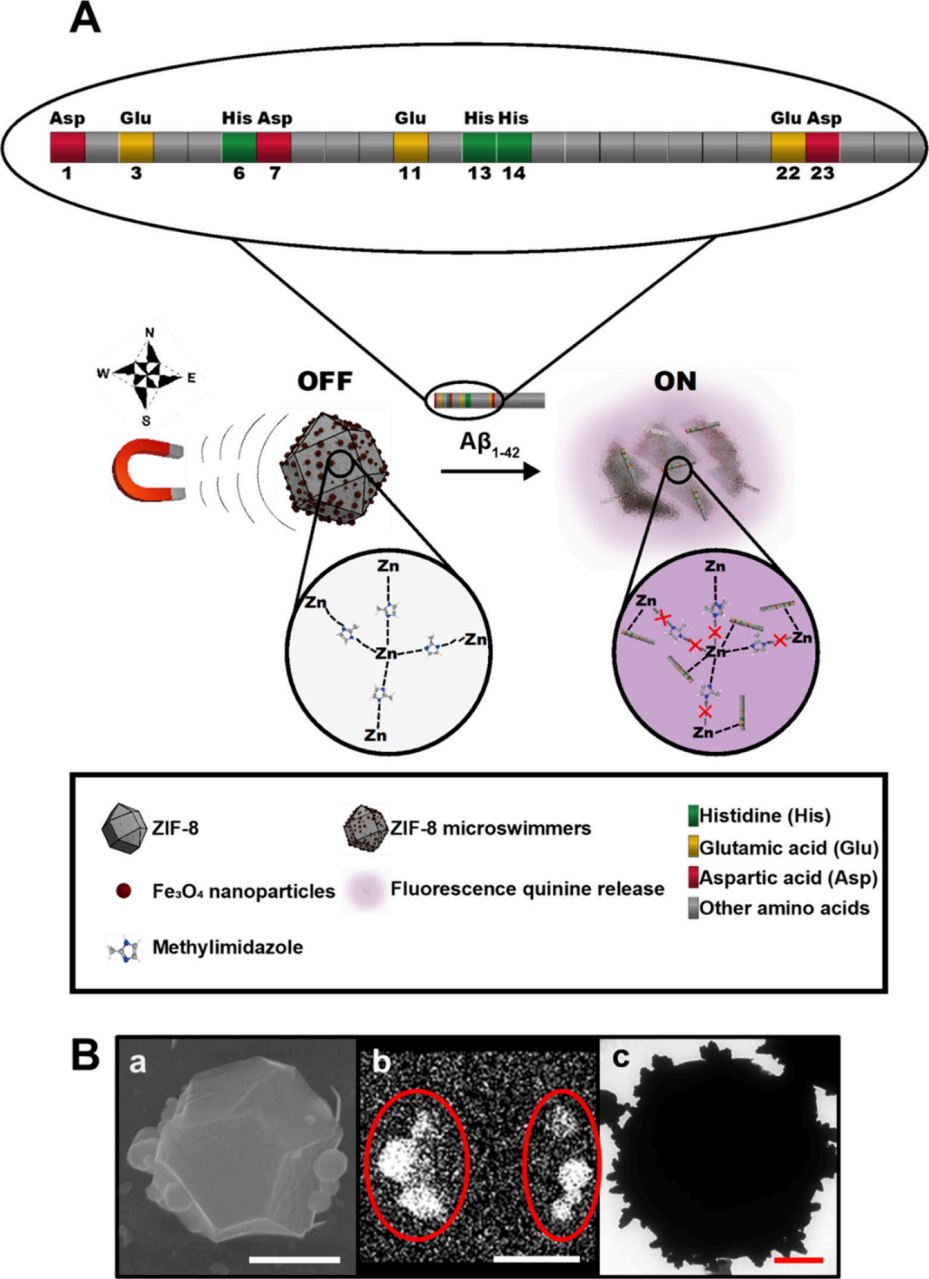
(A) Principle of the detection of Aβ_1–42_ peptide with macroporous ZIF-8 magnetic microswimmers. (B) (a) scanning-electrode microscopy (SEM) image of ZIF-8 microswimmer, (b) corresponding energy dispersive X-ray spectrometry (EDX) showing Fe distribution (as indicated with red circles), and (c) transmision electron microscopy (TEM) images. Scale bars, 1 μm.

Adequate microswimmer design and synthesis are key to obtaining the best analytical performance. The crystallization of ZIF-8 at room temperature can be modulated by using different ligands with different functionalities.^33^ In this work, a mix of 1-methylimidazole and 2-methylimidazole with a 4:1 ratio with respect to Zn^2+^ was used. Such a mix allows to obtain well-defined monocrystal-like structures at room temperature with a rhombic dodecahedron shape.^34,35^ The synthesis mechanism can be explained by the complex formation and ligand exchange, resulting in ZIF-8 with a length of about 2 μm in the long axis and positive surface charge due to the exposed Zn^2+^ ions on the external surface.^36^ The rhombic dodecahedron shape is preferred for further microswimmer fabrication due to an increase in the external surface area for the incorporation of a higher density of iron oxide nanoparticles for enhanced propulsion.^37,38^

For external decoration of the microswimmers with Fe_3_O_4_ nanoparticles, the positive nanoparticles^39^ were first modified with PSS, a polyelectrolyte that imparts them with a negative charge, for subsequent interaction with the positively charged ZIF-8 MOFs.^36,40^ The successful external decoration is reflected in the SEM and corresponding EDX images of Figure 1B (a, b, respectively) and the TEM image of Figure 1B, c. The X-ray difraction (XRD) spectra of Figure S1 reveals the successful generation of the ZIF-8 microswimmers, with the characteristic peaks (planes) at 2θ = 7.4° (011), 10.4° (002), 12.7° (112), 14.7° (022), 16.4° (013), 18.0° (222), 22.1° (114), 24.5° (233), 26.7° (134), and 29.6° (044).^35,41,42^ Quinine was chosen for encapsulation and detection due to the presence of methyl groups to attach to the methylimidazole groups in the ZIF-8 microswimmers via van der Waals forces^43^ as well as its analytical performance in terms of fluorescence stability and sensitivity. To improve the loading of quinine, during ZIF synthesis, we added to the mixture 100 nm PS nanoparticles to generate a macroporous structure.^44,45^ As the surface of PS nanoparticles is negatively charged, they can act as nucleation points of Zn^2+^ for subsequent assembly of ligands and ZIF-8 formation.^46^ The macroporous network is generated after the PS particles are dissolved with organic solvents. To demonstrate the as-claimed higher loading capacity, we prepared ZIF-8 microswimmers without adding PS particles (denoted as microporous) for comparison. The ex-situ quinine encapsulation protocol is detailed in the Experimental Section. DMF is used as an organic solvent because of its chemical properties, which allow the dissolution of quinine and the preservation of the ZIF-8 microswimmer structure during incubation. For the release, we use sulfuric acid at pH 2.0 to promote the microswimmer disassembly dissolution, fast quinine release, and to improve the quinine fluorescence signal.^47^ Experiments were conducted by resuspending 2 mg of each type of microswimmer in 1 mL of 0.01 M H_2_SO_4_ and placing 100 μL of the solutions in the well plate. The final fluorescence values plotted were obtained by subtracting the fluorescence values of ZIF-8 incubated without quinine as a control (n = 3). As can be seen in Figure 2, the macroporous microswimmers encapsulate approximately 50% more quinine as compared with microporous microswimmers, illustrating that the macroporous inner structure increased the surface area and, in turn, the loading capacity of the microswimmers. In addition, when quinine is not encapsulated in ZIF-8 microswimmers, the fluorescence is almost zero. This fact is further reflected in the time-lapse fluorescence microscopy images in Figure 2. Please note that the quinine fluorescence increases in acidic media. Note also the increase in the fluorescence intensity from the release experiments with macroporous microswimmers as compared with the microporous microswimmers, which is in line with the previous information.

**Figure 2:**
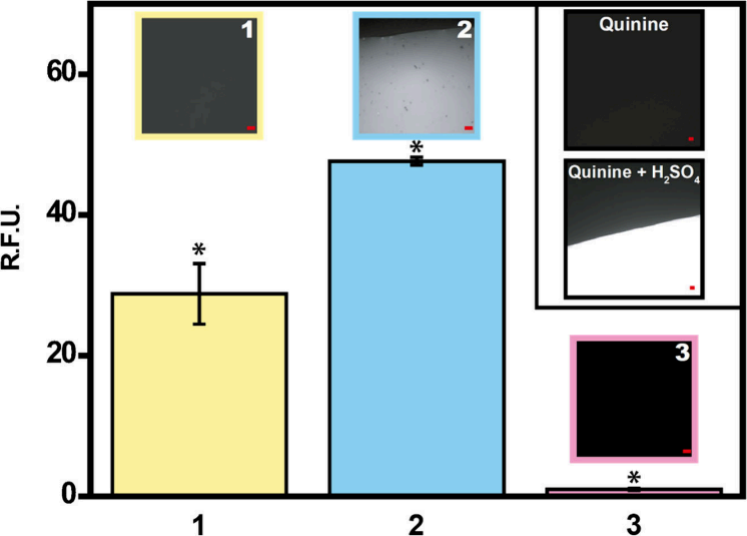
Quinine probe detection by fluorescence microscopy. Plot showing the fluorescence measured from released (1) microporous and (2) macroporous ZIF-8 microswimmers and (3) macroporous ZIF-8 microswimmers without quinine. *Statistically significant difference (*p* < 0.05). Error bars represent the mean values ± standard deviation (*n* = 3). Accordingly, images at the top of each bar correspond to the time-lapse fluorescence microscopy images (λ_ex_ = 385 nm, λ_em_ = 447 nm), while the inset shows the images of quinine hydrochloride (25 mM) and quinine hydrochloride (25 mM) + H_2_SO_4_ (0.01 M). Scale bars: 10 μm.

### Magnetic Characterization of ZIF-8-Based Microswimmers

To design ZIF-8 microswimmers with good magnetic capabilities for an adequate biosensing performance in low volumes of viscous CSF samples, critical variables were optimized. Indeed, the movement of the microswimmers helps to overcome viscosity issues in the complex samples, enhancing the mixing effect and allowing detection in microliters of sample. To this end, a fluid dynamics simulation was carried out to obtain information about the internal mixing of a drop containing ZIF-8 microswimmers (see the Experimental Section and Supporting Information for more details) decorated with 200 μg/mL Fe_3_O_4_ nanoparticles. Time-lapses of the corresponding images of the microswimmer propulsion at time 0 and after 2 min and of the flow dynamic simulations after 1 s of movement are depicted in Figure S2 and Video S1. The displacement was analyzed to model the motion using the CFD tool embedded in the Solidworks software (for more details, see the Experimental Section). Two components were taken into consideration for the flow dynamics simulation: rotational motion (0.39 Hz) and translational motion (0.47 μm/s), according to the experimental results. Simulations illustrate the flow dynamics around the microswimmers while actuated with the electromagnet system. In this case, by combining rotational and translational motion, the diffusion around the microswimmers is improved, revealing the crucial role of the magnetic actuation in the disassembly of ZIF-8 magnetic microswimmers disassembly.

Figure 3 shows in detail the studies of the magnetic composition and characterization in terms of motion and capabilities of the ZIF-8 magnetic microswimmers. Details of the calculation of the speed are given in the Experimental Section and in the Supporting Information.

**Figure 3:**
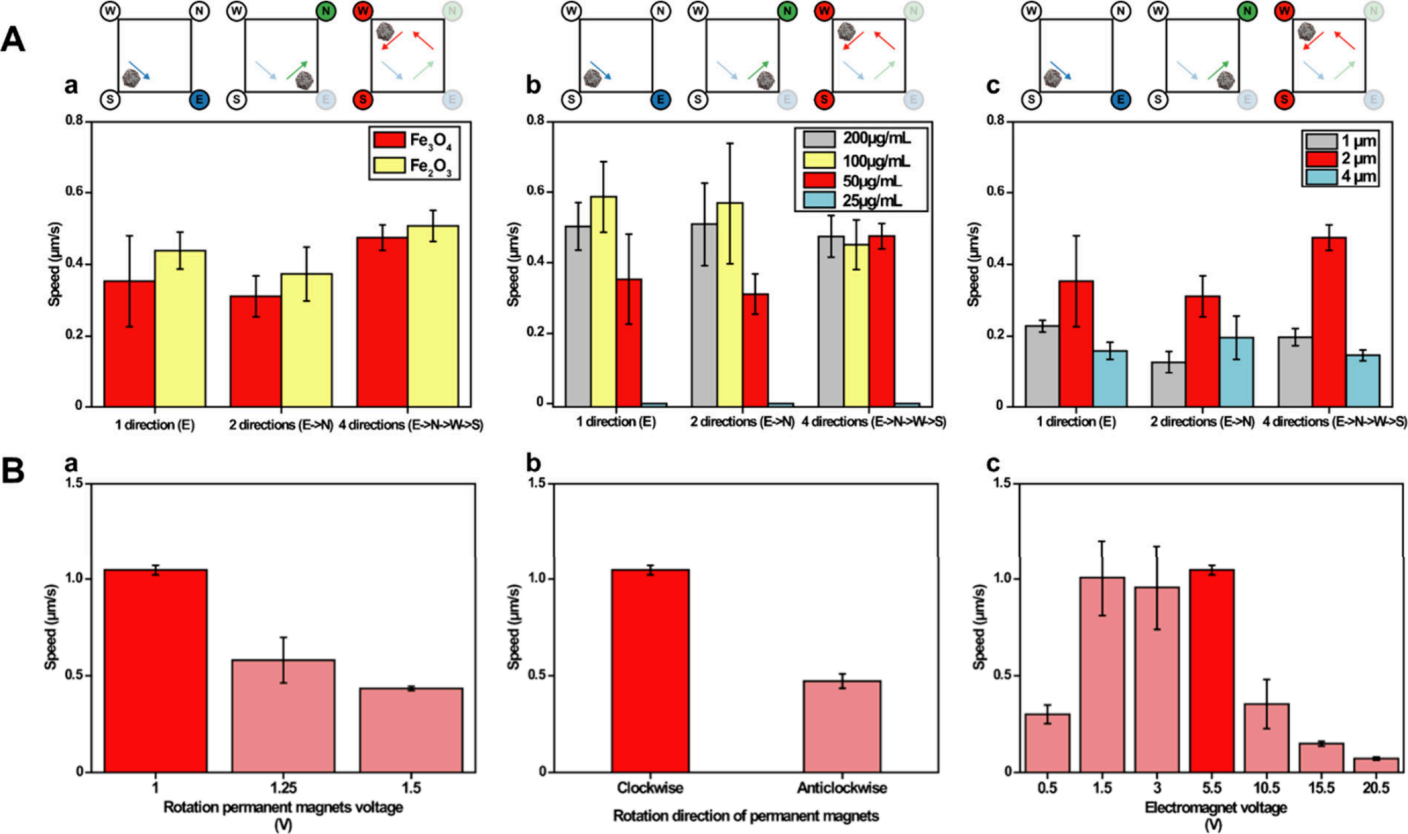
Optimization of the magnetic propulsion of ZIF-8 magnetic microswimmers. (A) Speed measurements of magnetic microswimmers in 1, 2, and 4 directions. For 1 directional motion, only the “E” electromagnet was activated. For 2 directional motions, “E” and “N” were sequentially activated (“E → N”), and for 4 directional motions, the sequence was “E → N → W → S”. In all experiments, a rotating permanent magnet remained active, and only one electromagnet was powered at any given time. Each directional change involved turning off the current electromagnet and immediately turning on the next one in sequence. The labels “E”, “N”, “W”, and “S” refer to the respective electromagnets positioned in those cardinal directions. (a) Microswimmers with different types of iron oxide nanoparticles (Conditions: ZIF-8 size = 2 μm, iron oxide concentration = 50 μg/mL, electromagnet voltage = 10.5 V, rotating permanent magnet voltage = 1 V and clockwise direction); (b) concentration of Fe_3_O_4_ nanoparticles (Conditions: ZIF-8 size = 2 μm, electromagnet voltage = 10.5 V, rotating permanent magnet voltage = 1 V and clockwise direction); and (c) ZIF-8 size (Conditions: Fe_3_O_4_ concentration = 50 μg/mL, electromagnet voltage = 10.5 V, rotating permanent magnet voltage = 1 V and clockwise direction). (B) Process of optimizing electromagnet system conditions to obtain optimum movement. Speed measurements of (a) microswimmers with different rotation permanent magnets voltage (Conditions: direction = N, ZIF-8 size = 2 μm, Fe_3_O_4_ concentration = 50 μg/mL, electromagnet voltage = 5.5 V and clockwise direction); (b) with different direction of rotation permanent magnets (Conditions: direction = N, ZIF-8 size = 2 μm, Fe_3_O_4_ concentration = 50 μg/mL, electromagnet voltage = 5.5 V and rotating permanent magnet voltage = 1 V); and (c) with different electromagnet voltages (Conditions: direction = N, ZIF-8 size = 2 μm, Fe_3_O_4_ concentration = 50 μg/mL, rotating permanent magnet voltage = 1 V and clockwise direction). Error bars represent the mean values ± standard deviation (*n* = 5).

First, we tested the influence of the composition, concentration, and size on the magnetic engine. To this end, the ZIF-8 microswimmers were modified with Fe_3_O_4_ and Fe_2_O_3_ nanoparticles. No differences in the linear speed were observed (see Figure 3A, a) in the 1, 2, and 4 directions allowed by the experimental setup. We also perform (3-[4,5-dimethylthiazol-2-yl]-2,5 diphenyl tetrazolium bromide) assay (MTT) with HeLa cells incubated with different concentrations of Fe_2_O_3_ and Fe_3_O_4_ nanoparticles. As can be seen in Figure S3, the viability of the cells is close to 100% (and statistically comparable with the control, α = 0.05) with Fe_3_O_4_ nanoparticles, as compared with the relatively lower viability (and statistically hindered biocompatibility, α = 0.05) of Fe_2_O_3_ nanoparticles, with viabilities from 80 to 85%. In addition, attending to the size of the nanoparticles tested, the Fe_3_O_4_ nanoparticles possess the optimal size (50–100 nm) vs Fe_2_O_3_ nanoparticles (<50 nm) for magnetization and hence, optimal propulsion properties.^48^ Consequently, Fe_3_O_4_ nanoparticles were chosen for microswimmers modification. Next, we optimized the concentration of Fe_3_O_4_ nanoparticles for the modification of the microswimmers. As can be seen in Figure 3A, b, no motion was observed using 25 μg/mL of the nanoparticles. At increasing concentrations, good motion and speed were observed. As a compromise among higher biocompatibility, more area exposed for encapsulation, and considering the motion in 4 directions (which will be consequently used to guide the microswimmers to the target sites), we chose 50 μg/mL as the optimal concentration. We also tested the influence of the size of the ZIF-8 microswimmer on the speed (Figure 3A, c). The higher speeds were noted when using 2 μm size microswimmers, with a drastic reduction with 4 μm sizes, probably due to the higher size of the microswimmer increasing the collisions with the glass slide, thus reducing the speed. Once we chose the optimal Fe_3_O_4_ microswimmers composition, we studied the magnetic motion and capabilities toward the influence of rotating magnets voltage, the rotation direction of the permanent magnets, and the electromagnet voltage, (Figure 3B, a-c). The best conditions (in terms of higher speed) were obtained using 5.5 and 1 V and clockwise direction, respectively. The combination of the rotating permanent magnet, which generates a rotating magnetic field that allows the energy friction limit to be broken and thereby initiates displacement, and the applied magnetic fields resulted in net translational displacement, generating an efficient and desired self-vortex effect in low sample volumes and improving analytical performance further.

### Analytical Performance and Analysis of CSF Samples

We next tested the suitability of the magnetic microswimmers for Aβ_1–42_ peptide detection and quantification in CSF samples from AD diagnosed patients at different stages of the disease. To this end, and to develop an approach with full analytical capabilities, the micromotors, designed with optimal magnetic capabilities previously optimized (Figure S2 and Video S1), were used as smart microswimmers in an external magnetic incubator system. Please note that due to the small sample volumes available, detection is performed by fluorescence microscopy, as discussed above. The results are shown in Figure 4. We first optimized the detection time (denoted as incubation time; for more details, please see the Experimental Section). The discrete fluorescence release plot is reflected in Figure 4A, which fitted to a zero-order kinetic model (F = −*k*_0_t + F_0_, R^2^ = 0.998 *p* < 0.05) with the highest release observed after 10 min, which was then selected as optimal. This is also seen in the SEM images of Figure 4B; microswimmer disintegration is observed after 10 min of navigation in the Aβ_1–42_ peptide solution. Next, the calibration performance was studied by using different solutions containing increased concentrations of the Aβ_1–42_ peptide. The calibration plot is depicted in Figure 4C, showing an excellent linear correlation coefficient of 0.9991. The limits of detection and quantification, calculated as 3 and 10 times the standard deviation of the intercept divided by the slope of the calibration, respectively, are 40 and 140 pg/mL. The linear range spans from 140 to 1200 pg/mL, requiring just 10 μL of sample per analysis. Intraday precision was evaluated for all concentrations used in the calibration studies, yielding excellent values of RSD < 2% (n = 3). Interday precision was assessed for the highest concentration of 1000 pg/mL, also showing very good values of RSD < 6% (n = 3).

**Figure 4:**
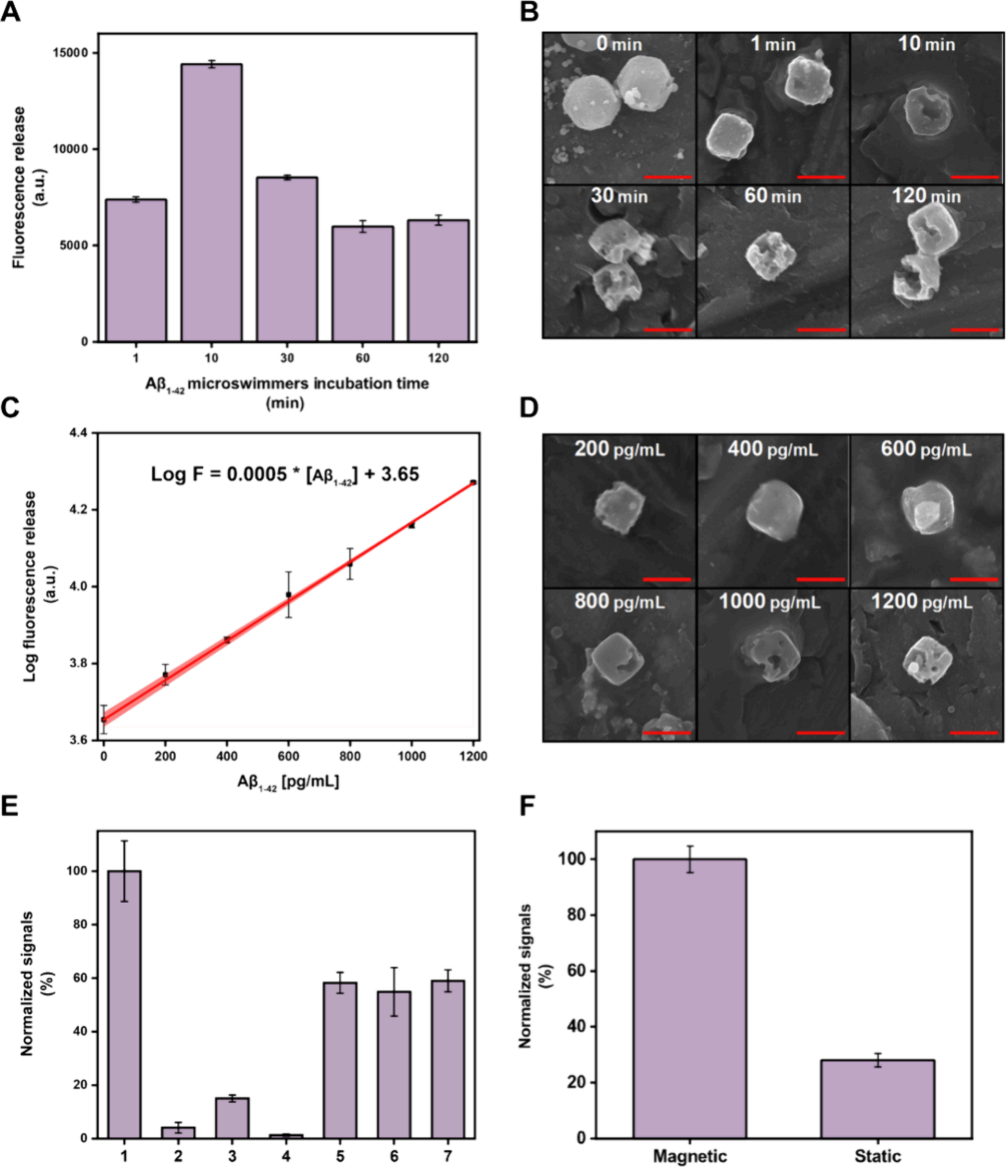
Aβ_1–42_ peptide detection and quantification with the quinine (25 mM) loaded ZIF-8 microswimmers. Optimization of the incubation time: (A) Fluorescence plots of the microswimmers before (0 min) and after incubation with 1000 pg/mL of the Aβ_1–42_ peptide for 1, 10, 30, 60, and 120 min and (B) corresponding SEM images. (C) Calibration plot microswimmers incubation with 200, 400, 600, 800, 1000, and 1200 pg/mL of Aβ_1–42_ and (D) corresponding SEM images. (E) Interference study in the presence of different coexisting proteins: 1: Aβ_1–42_ peptide [1000 pg/mL]; 2: Aβ_1–42_ oligomer [1000 pg/mL]; 3: Aβ_25–35_ fragment [1000 pg/mL]; 4: tau protein [1000 pg/mL]; 5: Aβ_1–42_ peptide [600 pg/mL]; 6: Aβ_1–42_ peptide + Aβ_1–42_ oligomer [600 + 600 pg/mL]; 7: Aβ_1–42_ peptide + tau protein [600 + 600 pg/mL]. (F) Role of the magnetic microswimmer movement in the detection. Error bars represent the mean values ± standard deviation (*n* = 3). Scale bars: 2 μm.

The SEM images of Figure 4D illustrate adequate destruction of the ZIF-8 microswimmers and that such destruction is more evident as the concentration of the target analyte increases, suggesting a higher release of quinine in the solution. Destruction is more evident at low concentrations when gaps first appear. However, it is difficult to see how the gaps grow in relation to lower concentrations when concentrations are higher.

Compared with other biosensors used for Aβ_1–42_ detection (see Table S1), our microswimmers allow detection in just 10 min, with the lowest LOD as compared with other fluorescence-based approaches. While other methods using electrochemical and especially electrochemiluminescence detection provide lower LODs, the main advantage of our approach is the reduction in the analysis time and the use of small sample volumes, holding considerable promise for future applications. Also, the linear range provided with our method allows for measurement within the level reported in the clinical practice, as will be further illustrated in the sample analysis. More importantly, most of the studies shown in Table S1 involved spiked samples, with only one measuring Aβ in real CSF samples. This highlights the value of our approach, which involves a significant number of samples, including those from healthy individuals and diagnosed patients.

The selectivity of our protocol was further evaluated by using common proteins that can be also present in AD samples, such as tau protein, Aβ_1–42_ oligomer, and Aβ_25–35_ fragment. Interestingly, as seen in Figure 4E, the selectivity is excellent, with a notable fluorescence signal only in the presence of the Aβ_1–42_ peptide. The absence of fluorescence in the presence of the Aβ_25–35_ fragment (C-terminal hydrophobic part) is attributed to the absence of the targeting amino acids that specifically bind to the Zn^2+^ to promote the disassembly of the microswimmers. Regarding tau protein, there is a controversy in the literature about potential binding sites to Zn^2+^. Tau structure consists on an unfolded protein with an acidic N-terminal region in the N-terminal part, a proline-rich region containing four repeat domains R1, R2, R3, and R4, and a C-terminal region.^49,50^ Cys in the repeat domains of tau (C291 and C322) has been identified as potential sizes for the Zn binding.^51^ In our case, however, no apparent interaction of the Zn^2+^ in the MOFs microswimmers is noted with tau protein, probably due to a lower affinity as compared to Aβ_1–42_ peptide or due to a folding of the protein used that prevents further interaction with the microswimmers. Indeed, the low dissociation constant (K_D_) of Aβ-Zn^2+^ is usually in the nM range, indicating a relatively high affinity.^52^ This means that Zn^2+^ binds strongly to Aβ, favoring its aggregation under physiological conditions. In the case of tau protein, the K_D_ of tau-Zn^2+^ is generally higher, (μM) range, at pathological concentration,^53,54^ similar to those found in AD neurodegeneration,^55^ indicating a lower affinity compared to Aβ. Also, to demonstrate the advantage of the use of microswimmers for dynamic detection, we perform control detection experiments in static. To this end, artificial CSF (ACSF) was fortified with 1000 pg/mL Aβ_1–42_ peptide. This suspension of microswimmers in ACSF was incubated under static or magnetic conditions (550 rpm) at 37 °C in dark, for 10 min. As illustrated in Figure 4F there is a 5-fold increase in the signal in moving vs static conditions, revealing the crucial role of the magnetic actuation and motion in the detection.

Once we evaluated the analytical performance of our approach, we applied the microswimmers for the analysis of CSF samples from healthy volunteers and patients previously diagnosed with mild cognitive impairment (MCI) or with AD. Demographic and clinical data of participants are listed in Table S2. First, we tested the efficient magnetic propulsion of the microswimmers in the ACSF and CSF samples (Figure 5A and B, Video S2). Efficient propulsion with a linear speed of 3.0 ± 0.6 μm/s (n = 5) was obtained in ACSF, whereas in CSF linear speed was 2.0 ± 0.4 μm/s (n = 5), illustrating the high towing force of the microswimmers in the samples for dynamic detection. These values demonstrate the high complexity of CSF samples, which contain a greater quantity of proteins and cellular debris than ACSF, which implies a speed decrease but does not hinder the capabilities of ZIF-8 microswimmers for analytical purposes.

**Figure 5:**
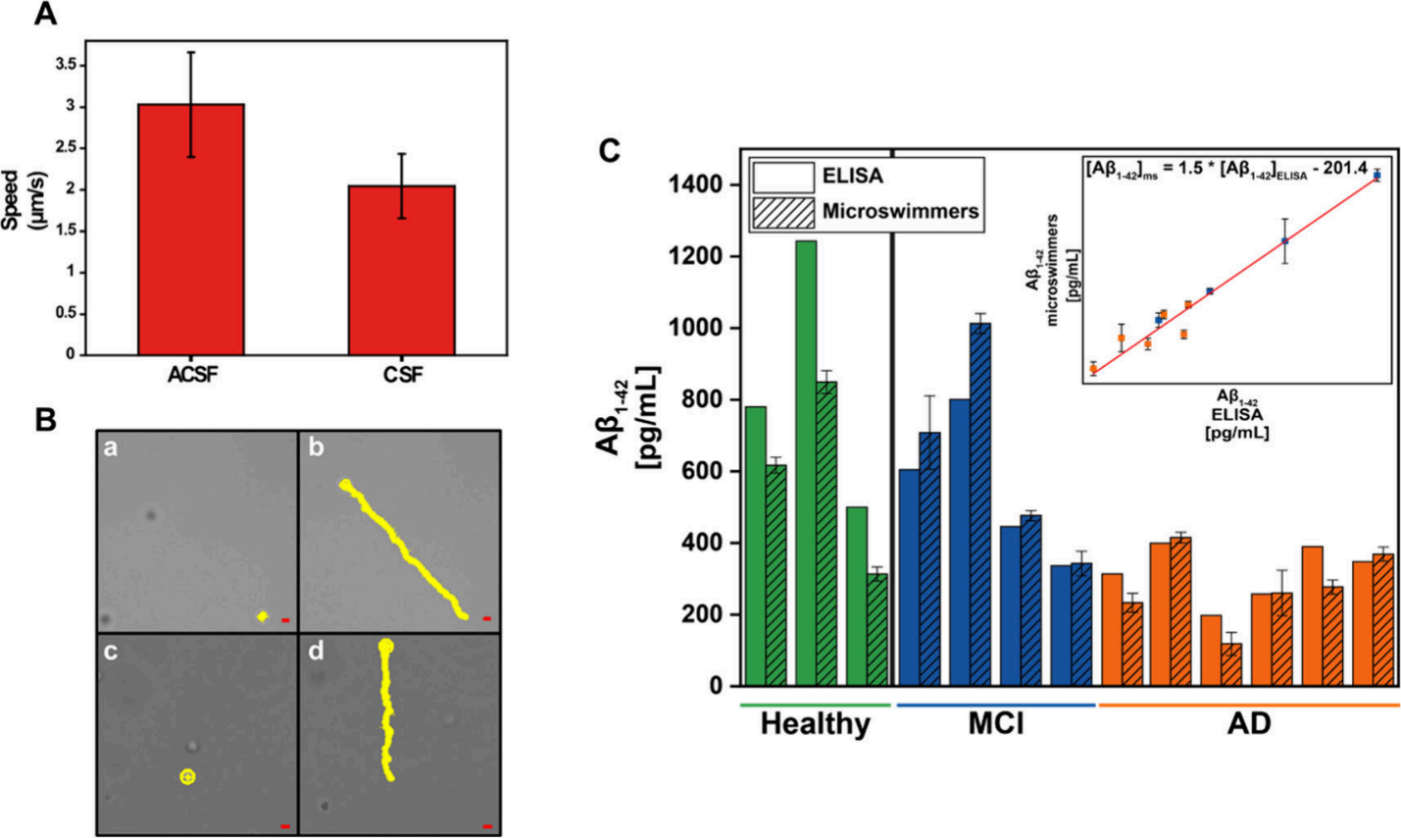
Quantification of the Aβ_1–42_ peptide and performance of the quinine-loaded ZIF-8 microswimmers in ACSF and CSF samples. (A) Speed of the microswimmers in ACSF and CSF samples. Error bars represent the mean values ± standard deviation (*n* = 5). (B) Time-lapse microcopy images (taken from Video S2) illustrating the microswimmer propulsion in ACSF samples (a) before and (b) after 30 s and in CSF samples (c) before and (d) after 30 s. Scale bars, 5 μm. (C) Plot summarizing the quantification of Aβ_1–42_ peptide with microswimmers after 10 min navigation in the CSF of patients and with ELISA as reference method. Error bars represent the mean values ± standard deviation (*n* = 3).

Finally, samples from healthy volunteers and AD diagnosed patients previously measured for Aβ_1–42_ peptide determination with the ELISA method were analyzed with the microswimmers. The results are shown in Figure 5C (see also Table S3 for the listed quantitative data). Interestingly, a very good correlation among the samples analyzed with the microswimmers and the ELISA values is obtained (r = 0.97). The SEM observation in Figure S4 illustrates the dissolution of the microswimmers in the AD samples (c-f) with less dissolution of the microswimmers in healthy samples (a,b) (yet some aggregation is noted), in line with the quantitative data obtained. It is very important to note that in the samples analyzed tau levels were also measured (see Table S2), indicating, as expected, the presence of this protein in the CSF samples as well. This confirms the good selectivity of the approach in the analysis of CSF samples, since no interference from the levels of this protein was inferred, maintaining the excellent correlation obtained. Although there are no levels established for Aβ_1–42_ peptide or total consensus in the literature, the good correlation of our microswimmer-based approach with the ELISA method, along with its simplicity, allows for the study and monitoring of such biomarkers in future studies.

## Conclusions

We have successfully developed quinine-encapsulated ZIF-8 magnetic microswimmers for self-fast destruction in just 10 min in only 10 μL of untreated CSF samples allowing the quantification of Aβ_1–42_ peptide as an amyloidosis biomarker for AD diagnosis. While no specific bioreceptors are used, the selectivity of the protocol is imparted by the affinity of a specific sequence of amino acids of Aβ_1–42_ peptide in the N-terminal hydrophilic part toward Zn^2+^ in the MOF structure. The excellent correlation obtained with the ELISA method in the healthy volunteers and diagnosed CSF analyzed samples from patients holds considerable promise as a less invasive approach, dramatically reducing the impact of the side effects of lumbar puncture. While antibodies and aptamers are widely used in clinical bioassays due to their high specificity, our microswimmers can be stored in a dried state for a long time without losing functionality or properties, and they can be used without any additional preparation. Moreover, they offer exceptional thermal and chemical stability, unlike bioreceptors that are sensitive to temperature and pH, which makes this approach an excellent alternative to those based on bioreceptors, which, although they exhibit exquisite selectivity, often involve higher costs, longer preparation times, and reduced stability under complex sample conditions.

Overall, this work reveals the potential of MOF-microswimmers as a novel bioreceptor-free approach to screening and predicting amyloidosis in the brain for AD diagnosis in the patient’s lifetime, opening the possibility of early treatment of the disease, as well as their potential as smart and elegant tools in clinical practice for monitoring not only for AD but also other neurodegenerative diseases.

## Supplementary Material







## Notes

Experimental section (Reagents and Materials, equipment, electromagnet system design and calculation of the speed of the microswimmers, flow dynamics simulation procedure; Supporting figures (S1–S4); Supporting Tables (S1–S3) (PDF)
Video S1 (MP4)
Video S2 (MP4)

## References

[1] Leite J. P., Figueira F., Mendes R. F., Almeida Paz F. A., Gales L. (2023). Metal-Organic Frameworks as Sensors for Human Amyloid Diseases. ACS Sens..

[2] Hardy J. A., Higgins G. A. (1992). Alzheimer’s
Disease: The Amyloid Cascade Hypothesis. Science.

[3] Scheltens P., Blennow K., Breteler M. M. B., de Strooper B., Frisoni G. B., Salloway S., Van der Flier W. M. (2016). Alzheimer’s
Disease. Lancet.

[4] Dubois B., Villain N., Frisoni G. B., Rabinovici G. D., Sabbagh M., Cappa S., Bejanin A., Bombois S., Epelbaum S., Teichmann M. (2021). Clinical Diagnosis of
Alzheimer’s Disease: Recommendations of the International Working
Group. Lancet Neurol..

[5] Jack C. R., Andrews J. S., Beach T. G., Buracchio T., Dunn B., Graf A., Hansson O., Ho C., Jagust W., McDade E. (2024). Revised Criteria for
Diagnosis and Staging of Alzheimer’s Disease: Alzheimer’s
Association Workgroup. Alzheimer's Dement..

[6] Ozin G. A., Manners I., Fournier-Bidoz S., Arsenault A. (2005). Dream Nanomachines. Adv. Mater..

[7] Mei Y., Solovev A. A., Sanchez S., Schmidt O. G. (2011). Rolled-up Nanotech
on Polymers: from Basic Perception to Self-Propelled Catalytic Microengines. Chem. Soc. Rev..

[8] Garcia-Gradilla V., Orozco J., Sattayasamitsathit S., Soto F., Kuralay F., Pourazary A., Katzenberg A., Gao W., Shen Y., Wang J. (2013). Functionalized
Ultrasound-Propelled Magnetically Guided Nanomotors:
Toward Practical Biomedical Applications. ACS
Nano.

[9] Wang, J. Nanomachines: Fundamentals and Applications; Wiley, 2013.

[10] Wang W., Duan W., Ahmed S., Mallouk T. E., Sen A. (2013). Small Power:
Autonomous Nano- and Micromotors Propelled by Self-Generated Gradients. Nano Tod..

[11] Esteban-Fernández
de Ávila B., Angsantikul P., Li J., Gao W., Zhang L., Wang J. (2018). Micromotors Go In Vivo: From Test
Tubes to Live Animals. Adv. Function. Mater..

[12] Pacheco M., López M. Á., Jurado-Sánchez B., Escarpa A. (2019). Self-Propelled Micromachines
for Analytical Sensing:
a Critical Review. Anal. Bioanal. Chem..

[13] Maria-Hormigos R., Jurado-Sánchez B., Escarpa A. (2022). Biocompatible Micromotors
for Biosensing. Anal. Bioanal. Chem..

[14] Cai L., Xu D., Zhang Z., Li N., Zhao Y. (2023). Tailoring Functional
Micromotors for Sensing. Res..

[15] Escarpa A., Jurado-Sánchez B. (2025). Micromotors
Meet Collective (Bio)­sensing:
The Asset Behind the Assay. Anal. Chem..

[16] Gordón
Pidal J. M., Moreno-Guzmán M., Montero-Calle A., Valverde A., Pingarrón J. M., Campuzano S., Calero M., Barderas R., López M. Á., Escarpa A. (2024). Micromotor-based Electrochemical Immunoassays for Reliable
Determination of Amyloid-β (1–42) in Alzheimer’s
Diagnosed Clinical Samples. Biosens. Bioelectron..

[17] Gordón
Pidal J. M., Moreno-Guzmán M., Montero-Calle A., Barderas R., López M. Á., Escarpa A. (2025). Dual On-The-Move Electrochemical
Immunoassays for the Simultaneous Determination of Amyloid-β
(1–42) and Tau in Alzheimer’s Patient Samples. Sens. Actuat. B. Chem..

[18] Gallo-Orive Á., Moreno-Guzmán M., Sanchez-Paniagua M., Montero-Calle A., Barderas R., Escarpa A. (2024). Gold Nanoparticle-Decorated
Catalytic Micromotor-Based Aptassay for Rapid Electrochemical Label-Free
Amyloid-β42 Oligomer Determination in Clinical Samples from
Alzheimer’s Patients. Anal. Chem..

[19] Jiao L., Seow J. Y. R., Skinner W. S., Wang Z. U., Jiang H.-L. (2019). Metal-Organic
Frameworks: Structures and Functional Applications. Mater. Tod..

[20] Khezri B., Pumera M. (2019). Metal-Organic Frameworks Based Nano/Micro/Millimeter-Sized
Self-Propelled Autonomous Machines. Adv. Mater..

[21] Terzopoulou A., Nicholas J. D., Chen X.-Z., Nelson B. J., Pané S., Puigmartí-Luis J. (2020). Metal-Organic
Frameworks in Motion. Chem. Rev..

[22] Bujalance-Fernández J., Jurado-Sánchez B., Escarpa A. (2023). The Rise of Metal-Organic
Framework Based Micromotors. Chem. Commun..

[23] Ikezoe Y., Washino G., Uemura T., Kitagawa S., Matsui H. (2012). Autonomous
Motors of a Metal-Organic Framework Powered by Reorganization of Self-Assembled
Peptides at Interfaces. Nat. Mater..

[24] Zhang G., Shen Y., Phipps J., Sun L., Ma S. (2024). Metal-Organic
Frameworks for the Diagnosis and Treatment of Alzheimer’s Disease:
Current Status and Perspectives. Coord. Chem.
Rev..

[25] Qin J., Cho M., Lee Y. (2019). Ferrocene-Encapsulated
Zn Zeolitic Imidazole Framework
(ZIF-8) for Optical and Electrochemical Sensing of Amyloid-β
Oligomers and for the Early Diagnosis of Alzheimer’s Disease. ACS Appl. Mater. Interfaces.

[26] Deng J., Wang K., Wang M., Yu P., Mao L. (2017). Mitochondria
Targeted Nanoscale Zeolitic Imidazole Framework-90 for ATP Imaging
in Live Cells. J. Am. Chem. Soc..

[27] Bujalance-Fernández J., Carro E., Jurado-Sánchez B., Escarpa A. (2024). Biocatalytic
ZIF-8 Surface-Functionalized Micromotors Navigating in the Cerebrospinal
Fluid: Toward Alzheimer Management. Nanoscale.

[28] McDade E., Wang G., Gordon B. A., Hassenstab J., Benzinger T. L. S., Buckles V., Fagan A. M., Holtzman D. M., Cairns N. J., Goate A. M. (2018). Longitudinal
Cognitive
and Biomarker Changes in Dominantly Inherited Alzheimer Disease. Neurology.

[29] Jack C. R., Bennett D. A., Blennow K., Carrillo M. C., Dunn B., Haeberlein S. B., Holtzman D. M., Jagust W., Jessen F., Karlawish J. (2018). NIA-AA Research Framework:
Toward a biological
definition of Alzheimer’s disease. Alzheimers
Dement..

[30] Sturchio A., Dwivedi A. K., Young C. B., Malm T., Marsili L., Sharma J. S., Mahajan A., Hill E. J., Andaloussi S. E. L., Poston K. L. (2021). High Cerebrospinal Amyloid-β 42
is Associated with Normal Cognition in Individuals with Brain Amyloidosis. eClinicalMed..

[31] Albert M. S., DeKosky S. T., Dickson D., Dubois B., Feldman H. H., Fox N. C., Gamst A., Holtzman D. M., Jagust W. J., Petersen R. C. (2011). The Diagnosis of Mild Cognitive Impairment
due to Alzheimer’s Disease: Recommendations from the National
Institute on Aging-Alzheimer’s Association Workgroups on Diagnostic
Guidelines for Alzheimer’s Disease. Alzheimers
Dement..

[32] Alies B., Conte-Daban A., Sayen S., Collin F., Kieffer I., Guillon E., Faller P., Hureau C. (2016). Zinc­(II) Binding
Site
to the Amyloid-β Peptide: Insights from Spectroscopic Studies
with a Wide Series of Modified Peptides. Inorg.
Chem..

[33] Cravillon J., Nayuk R., Springer S., Feldhoff A., Huber K., Wiebcke M. (2011). Controlling Zeolitic Imidazolate
Framework Nano- and
Microcrystal Formation: Insight into Crystal Growth by Time-Resolved
In Situ Static Light Scattering. Chem. Mater..

[34] Cravillon J., Münzer S., Lohmeier S.-J., Feldhoff A., Huber K., Wiebcke M. (2009). Rapid Room-Temperature
Synthesis and Characterization
of Nanocrystals of a Prototypical Zeolitic Imidazolate Framework. Chem. Mater..

[35] Zhang Y., Jia Y., Li M., Hou L. a. (2018). Influence of the 2-Methylimidazole/Zinc
Nitrate Hexahydrate Molar Ratio on the Synthesis of Zeolitic Imidazolate
Framework-8 Crystals at Room Temperature. Sci.
Rep..

[36] Oh S., Lee S., Lee G., Oh M. (2023). Enhanced Adsorption Capacity of ZIF-8
for Chemical Warfare Agent Simulants Caused by its Morphology and
Surface Charge. Sci. Rep..

[37] Suresh K., Kalenak A. P., Sotuyo A., Matzger A. J. (2022). Metal-Organic Framework
(MOF) Morphology Control by Design. Chem.—Eur.
J..

[38] Łuczak J., Kroczewska M., Baluk M., Sowik J., Mazierski P., Zaleska-Medynska A. (2023). Morphology Control Through the Synthesis of Metal-Organic
Frameworks. Adv. Colloid Interface Sci..

[39] Yu H., Li Y., Li X., Fan L., Yang S. (2014). Highly Dispersible
and Charge-Tunable Magnetic Fe_3_O_4_ Nanoparticles:
Facile Fabrication and Reversible Binding to GO for Efficient Removal
of Dye Pollutants. J. Mater. Chem. A.

[40] Wang S., Ye H., Wang Y., Ma X. (2022). Metal-Organic-Framework based Catalytic
Micromotor for Enhanced Water Decontamination. Chem. Select.

[41] Pan Y., Liu Y., Zeng G., Zhao L., Lai Z. (2011). Rapid Synthesis of
Zeolitic Imidazolate Framework-8 (ZIF-8) Nanocrystals in an Aqueous
System. Chem. Commun..

[42] Park K. S., Ni Z., Côté A. P., Choi J. Y., Huang R., Uribe-Romo F. J., Chae H. K., O’Keeffe M., Yaghi O. M. (2006). Exceptional Chemical
and Thermal Stability of Zeolitic
Imidazolate Frameworks. Proc. Nat. Acad. Sci..

[43] Liédana N., Galve A., Rubio C., Téllez C., Coronas J. (2012). CAF@ZIF-8: One-Step Encapsulation of Caffeine in MOF. ACS Appl. Mater. Interfaces.

[44] Zhou H., Zou Z., Dai L., Liu D., Du W. (2022). Ordered Macro-Microporous
ZIF-8 with Different Macropore Sizes and Their Stable Derivatives
for Lipase Immobilization in Biodiesel Production. ACS Sus. Chem. Eng..

[45] Zhang T., Zhang X., Yan X., Kong L., Zhang G., Liu H., Qiu J., Yeung K. L. (2013). Synthesis of Fe_3_O_4_@ZIF-8 Magnetic
Core-Shell Microspheres and Their Potential
Application in a Capillary Microreactor. Chem.
Eng. J..

[46] Xu H., Casabianca L. B. (2020). Probing
Driving Forces for Binding Between Nanoparticles
and Amino Acids by Saturation-Transfer Difference NMR. Sci. Rep..

[47] Eastman J. W. (1967). Quantitative
Spectrofluorimetry-the Fluorescence Quantum Yield of Quinine Sulfate. Photochem. Photobiol..

[48] Li Q., Kartikowati C. W., Horie S., Ogi T., Iwaki T., Okuyama K. (2017). Correlation
Between Particle Size/Domain Structure
and Magnetic Properties of Highly Crystalline Fe_3_O_4_ Nanoparticles. Sci. Rep..

[49] Mandelkow E.-M., Mandelkow E. (2012). Biochemistry
and Cell Biology of Tau Protein in Neurofibrillary
Degeneration. Cold Spring Harb. Perspec. Med..

[50] Martin L., Latypova X., Wilson C. M., Magnaudeix A., Perrin M.-L., Yardin C., Terro F. (2013). Tau protein
kinases:
Involvement in Alzheimer’s disease. Ag.
Res. Rev..

[51] La
Rocca R., Tsvetkov P. O., Golovin A. V., Allegro D., Barbier P., Malesinski S., Guerlesquin F., Devred F. (2022). Identification of the Three Zinc-Binding Sites on Tau
Protein. Int. J. Biol. Macromol..

[52] August A., Hartmann S., Schilling S., Müller-Renno C., Begic T., Pierik A. J., Ziegler C., Kins S. (2024). Zinc and Copper
Effect Mechanical Cell Adhesion Properties of the Amyloid Precursor
Protein. Biol. Chem..

[53] Mo Z.-Y., Zhu Y.-Z., Zhu H.-L., Fan J.-B., Chen J., Liang Y. (2009). Low Micromolar Zinc
Accelerates the Fibrillization of Human Tau via
Bridging of Cys-291 and Cys-322. J. Biol. Chem..

[54] Moreira G. G., Cristóvão J. S., Torres V. M., Carapeto A. P., Rodrigues M. S., Landrieu I., Cordeiro C., Gomes C. M. (2019). Zinc Binding
to Tau Influences Aggregation Kinetics and Oligomer Distribution. Int. J. Mol. Sci..

[55] Sensi S. L., Granzotto A., Siotto M., Squitti R. (2018). Copper and Zinc Dysregulation
in Alzheimer’s Disease. Trends Pharmacol.
Sci..

