# Supplementary material for: ZIF‑8 Microswimmers Self-Fast Dynamic Destruction in Microliter Volumes of Cerebrospinal Fluid Samples: Toward a Selective Assessment of Amyloidosis
Source: Anal Chem. 2025 Aug 15;97(33):18282–91. doi: 10.1021/acs.analchem.5c03467 (PMC12392251; doi:10.1021/acs.analchem.5c03467)
Supplement: Supplementary file 1 [file ac5c03467_si_001.pdf]

ZIF-8 microswimmers self-fast dynamic destruction in microliter volumes of cerebrospinal fluid samples: toward a selective assessment of amyloidosis

Javier Bujalance-Fernández,[a] Eva Carro,[b,c] Desiree Antequera,[b,c] Beatriz Jurado-Sánchez,[a,d] Alberto Escarpa [a,d]**

[a]Department of Analytical Chemistry, Physical Chemistry and Chemical Engineering, Universidad de Alcala, Alcala de Henares, E-28802 Madrid, Spain

[b]Chronic Disease Programme, UFIEC, Carlos III Health Institute, E-28029, Majadahonda, Madrid, Spain

[c]CIBERNED, E-20031, Madrid, Spain

[d]Chemical Research Institute "Andres M. del Rio", Universidad de Alcala, E-28802, Madrid, Spain

TABLE OF CONTENTS

-Experimental section……………………………………………………………..S2

-Supporting figures…………………………………………………………...…S5

-Supporting tables.………………………………………………………...……S8

-References……...………………………………………………………..…S11

EXPERIMENTAL SECTION

Reagents and Materials. Hydrophilic polytetrafluoroethylene membranes of 0.1 μm pores and 13 mm of diameter (cat. JVWP01300) and anodisc inorganic filter membranes of 0.2 μm pores, 25 mm of diameter (cat. WHA68096022), Amyloid beta 42 reference protein in CSF (cat. ERM-DA482_IFCC) and Amyloid beta protein fragment 25-35aa (cat. A4559) were purchased from Merck Millipore (Spain). Methanol (cat. 34860), 1-Methylimidazole (cat. M50834), 2-Methylimidazole (cat. M50850), zinc nitrate hexahydrate (cat. 96482), N,N-dimethylformamide (DMF) (cat. 543897), iron (III) oxide nanopowder (cat. 544884), quinine hydrochloride dihydrate (cat. 8.22194) and micro particles 100nm based on polystyrene (cat. 43302) were purchased from Sigma-Aldrich (Madrid, Spain). Glass microscopic slides (cat. 12302158), cyclopore polycarbonate membranes of 0.2 μm pores and 25 mm of diameter (cat. 11374814), iron (II, III) oxide nanopowder (cat. 047141.36), sulfuric acid (cat. 12656777), nitric acid (cat. 10050270) and hydrochloric acid (cat. 0000180) were purchased from Fisher Scientific (Madrid, Spain). Protein LoBind Tubes 1.5mL (cat. 0030108116) were purchased from Eppendorf (Madrid, Spain). Amyloid beta 42 oligomeric (cat. PE-1750-1000) and Human cerebral spinal fluid (pooled donors) (cat. 088T-CSFP) were purchased from Tebu-Bio (Spain). Recombinant Human Tau-441 (2N4R) (cat. 842501) was purchased from BioLegend. Artificial Cerebrospinal Fluid (ACSF, cat. 3525/25ML) was purchased from Biotechne. Target2 Nylon Syringe Filters (cat. F2504-2) was purchased from ThermoFisher Scientific. 1mL syringe (cat. MDSS01SE) was purchased from (Terumu Pharmaceutical Solutions). Milli Q water was obtained using a Millipak Express Filter (cat. MPGP04001) and Vent Filter (cat. TANKMPK01) were purchased from Merck Millipore (Madrid, Spain). All reagents were used without further purification.

Equipment. A DynaMagTM-2 magnetic rack (cat. 12321D) was used to hold and wash the microswimmers. An ultrasonic bath (Elmasonic S 30 H) (cat. 100 1955) and a probe CV18 attached to an ultrasonic processors Vibra-Cell™ VCX 130 were used to carry out the ZIF-8

magnetization. An Eppendorf Centrifuge 5430 attached with an FA-45-30-11 rotor was used for cleaning steps and materials synthesis. An inverted Nikon Eclipse Ti–S/L100 optical microscope, coupled with Zyla sCMOS camera, was used to capture images and record videos. The speed of the microswimmers was recorded and measured using NIS-5.41 elements software. The microscope is equipped with a LED light source (CoolLED's pE-4000, CoolLED Ltd, UK) attached and a DAPI-5060C (λ_{ex} 377/50 nm, λ_{em} 447/60) filter cube (Nikon). microswimmers speed was recorded and measured using NIS-5.41 elements software. BioTek Cytation 5 Cell Imaging Multimode Reader was used for fluorescence measurements. SEM characterization of the microswimmers was performed using a JEOL JSM 6335F microscope coupled to Xflash detector 4010 (Bruker); and a JEOL STEM JSM-IT500 microscope. TEM microscopy (Zeiss EM10C) was utilized to capture images of ZIF-8 and microswimmers. Eppendorf ThermoMixer™ C was used as an incubator to obtain macroporous ZIF-8 and quinine loading. IKA KS 3000i Control was used as an incubator to produce the quinine release from ZIF-8 microswimmers. A Stamos Soldering S-LS-75 Laboratory Power Supply was used as a source of energy to the magnetic system. 3-[4,5-Dimethylthiazol-2-yl]-2,5-diphenyltetrazolium bromide (MTT) assays were performed in the Cell Culture Centre of the Universidad de Alcala. The results were obtained from 6 replicates of HeLa cell incubation under different conditions.

Electromagnet system design and calculation of the speed of the microswimmers. The system was assembled by using Heschen electromagnet solenoids (small system: P30/22, 30 mm, 12V or big system: P50/27, 50 mm, 24V). An Arduino Nano board was used to switch the relays on demand and the serial port of the microcontroller was used as a user interface to input the commands corresponding to the desired magnetic gradient direction. A stage to hold all the magnetic elements was designed using the SolidWorks CAD software and it was printed using an Original Prusa i3 MK3S+ printer. To record the videos using the tailor-made device, the magnetic ZIF-8 microswimmers were magnetized for 10 seconds followed by 10 seconds of ultrasound in a bath, and then 4 μL of the solution was dropped onto a slide and recorded with the camera attached to the optical microscope. To obtain videos that could be compared between them, each was recorded with the same duration. In all cases, the video began with 5 seconds of static, followed by 10 seconds of only the activation of the rotating permanent

magnets, and finally, 2 minutes of 1 electromagnet activation (in the case of 1 direction), 1 minute of 1 electromagnet followed by 1 minute of a perpendicular electromagnet (in the case of 2 directions), or 30 seconds of each one of the magnets perpendicularly activating the next one (in the case of 4 directions). In all cases, the total duration of each video was 2 minutes and 15 seconds. The voltage of the magnets depends on the different conditions applied in each study. To measure the microswimmer speed, a coordinate where the microswimmer is located at time 0 was used as reference "A" and a coordinate after the movement performed in a direction at time X was used as reference "B". As such, it was possible to calculate the displacement produced between point A and point B and divide it by the exact time taken to travel from point A to point B, thus obtaining an exact speed for each of the directions. We adopted this approach since the automatic tracking software of the microscope considers the rotation of the microswimmer (induced by the permanent magnet) as speed, but this is wrong since no actual displacement is produced. To calculate the speed, the displacement (Δx) was calculated as Euclidean distance $(\mathrm{Dx})^{2} = \mathrm{a}^2 + \mathrm{b}^2$, with a small modification $(\mathrm{Dx})^{2} = (\mathrm{b}_1 - a_1)^2 + (b_2 - a_2)^2$ where "(b_1-a_1)" is the distance that the microswimmer has displaced in the abscissa axis, "(b_2-a_2)" is the distance that has displaced in the ordinate axis and "(Δx) " is the displacement produced by the microswimmer between the reference "A" and "B". The displacement time is calculated according to the formula $\mathrm{t} = \mathrm{n} \cdot \mathrm{k}$, where "t" is the total time of the movement in seconds, "n" is the number of frames of the movement and "k" is the time each frame lasts, which is a constant = 0.0245 s. The velocity in one direction was calculated as $v = \frac{(\mathrm{Dx})}{t}$, where "v" is the velocity of the microswimmer in the movement in one direction. The total speed of the microswimmer was calculated as follows:

$$v_T = \frac{\sum_{i=1}^{4}(v_i \cdot n_i)}{\sum_{i=1}^{4} n_i}$$

Where **"v_T"** corresponds to the weighted average of the speeds of the different directions. Please note here the significant differences in the speed of our microswimmers, discarding thus Brownian motion. First, Brownian motion has a random displacement in any direction and changes direction in a random time, whereas our motion is linear in the direction we choose throughout the recording time. If the motion were Brownian, the final displacement would be theoretically 0, whereas our displacement is greater than zero in the range of a few μm per

second. Notably, Brownian motion was not observed, even when the electromagnetic system was inactive, probably due to the microswimmers' size and geometry.[1,2]

Flow dynamics simulation procedure. CFD tool embedded in the Solidworks software was used to model the motion. Motion was recorded in only one direction under 1V on the rotating permanent magnets and 10.5V voltage supply on the electromagnets. Two components were taken into consideration for the flow dynamics simulation: rotational motion (0.39 Hz), and translational motion (0.47 μm/s), according to the experimental results. Simulations illustrate the flow dynamics around the microswimmer while actuated with the electromagnet system. In this case, by combining rotational and translational motion, the diffusion around the microswimmer is improved.

Kinetic model of quinine release evaluation. Data from the quinine encapsulation and release for $A\beta_{1-42}$ quantification experiments was analyzed according to the zero-order kinetic model.

SUPPORTING FIGURES

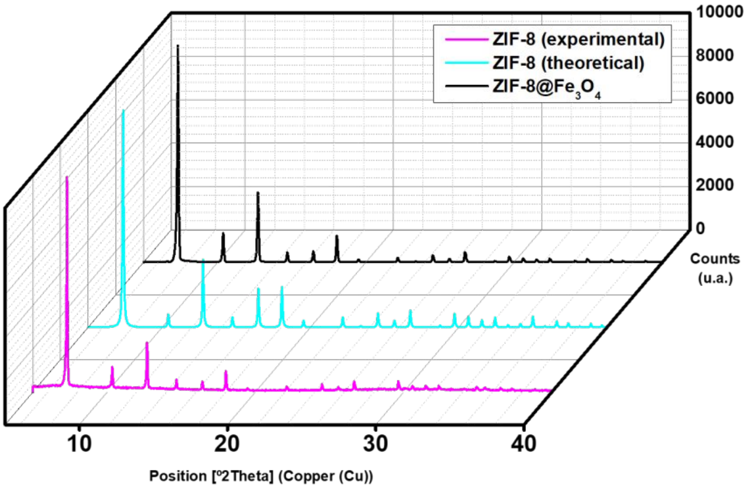

Figure S1. Experimental XRD spectrum of ZIF-8 and ZIF-8 externally decorated with Fe_3O_4 and theoretical XRD spectrum of ZIF-8.

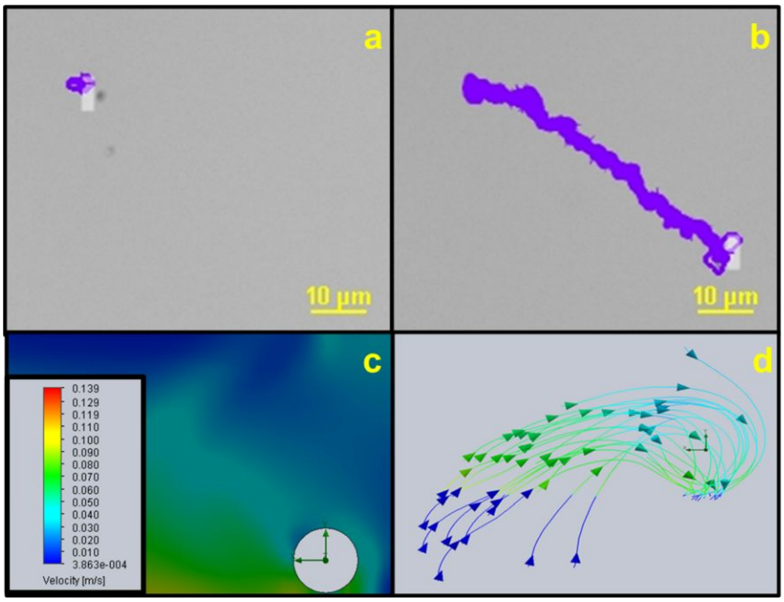

Figure S2. Time-lapse microscopy images, at (a) time 0 and (b) after 2 minutes, and flow dynamic simulations (taken from **Video S1**) of (c) the Y- and X-axis sections and (d) the back view trajectories of the ZIF-8 microswimmers. The corresponding boundary and initial conditions were applied, namely, a linear velocity of 0.47 μm/s in the X-axis and an angular frequency (w) of 0.39 Hz around the Y-axis were considered according to the experimental observations in the steady state. Other conditions, ZIF-8 size: 5 μm, Fe_3O_4 concentration: 200 μg/mL, electromagnet voltage: 10.5 V, rotating permanent magnet voltage: 1 V and clockwise direction. Scale bars: 10μm.

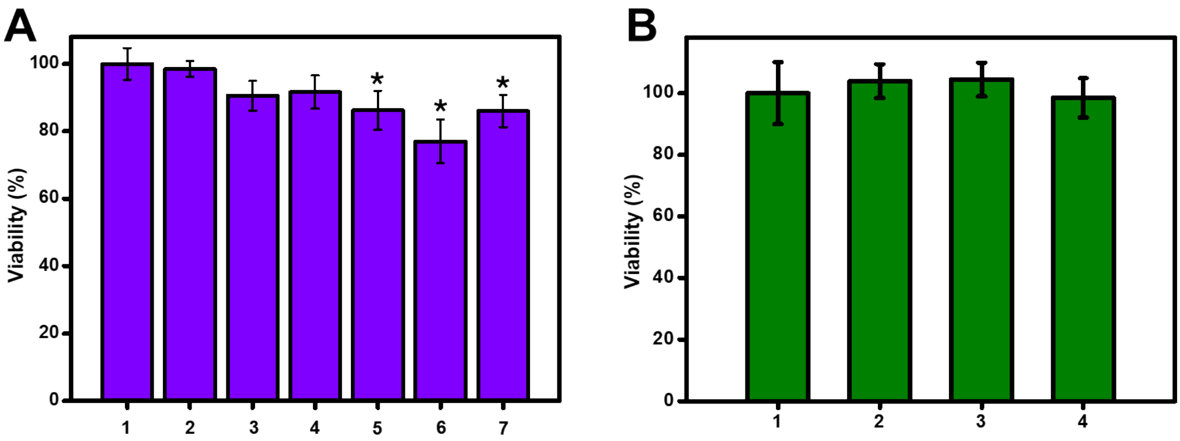

Figure S3. (A) MTT assays obtained from HeLa cell incubation where: 1) Untreated control; 2) 200 μg/mL Fe_3O_4 nanoparticles; 3) 100 μg/mL Fe_3O_4 nanoparticles; 4) 50 μg/mL Fe_3O_4 nanoparticles; 5) 200 μg/mL Fe_2O_3 nanoparticles; 6) 100 μg/mL Fe_2O_3 nanoparticles; 7) 50

μg/mL Fe_2O_3 nanoparticles. Error bars represent the standard deviation of 6 measurements. (B) MTT assays obtained from HeLa cell incubation where: 1) Untreated control; 2) 600.000 ZIF-8 with external 50 μg/mL Fe_3O_4 nanoparticles; 3) 300.000 ZIF-8 with external 50 μg/mL Fe_3O_4 nanoparticles and 4) 60.000 ZIF-8 with external 50 μg/mL Fe_3O_4 nanoparticles. Error bars represent the standard deviation of 6 measurements. *Statistically significant difference from untreated control condition.

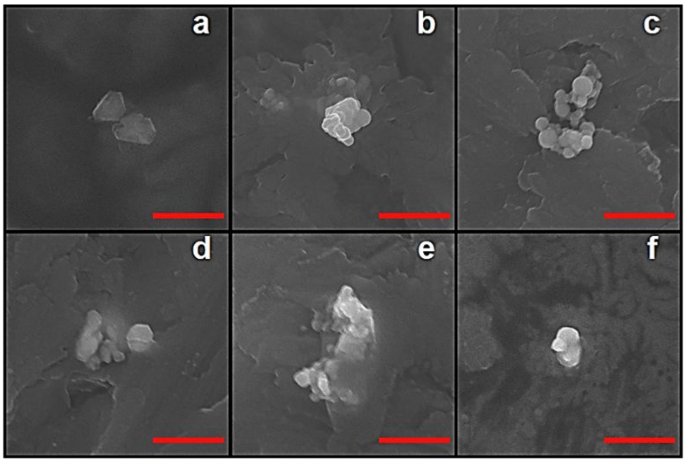

Figure S4. SEM images of the microswimmers after 10 minutes navigation on CSF samples from healthy patients (a) C1 and (b) C2 and AD patients at different stages of the illness (c) P2, (d) P4, (e) P7 and (f) P10. Scale bars, 2 μm.

Table S1. Comparison of the analytical features of the ZIF-8 microswimmers with other sensors for *$A\beta_{1-42}$* detection.

Sensing approach	Detection principle	LOD (pg/mL)	Sample volume	Time (minutes)	Sample analysis	Ref.
Sandwich antibody using ferrocene immobilized ZIF-8 as tag	Electrochemical	0.03	25 µL	120	Fortified serum	1
Antibody modified polypirrol/Pt catalytic micromotors	Electrochemical	60	25 µL	15	Tissue, plasma and CSF	2
Antibody-modified SnO_2 nanofibers	Electrochemical	1.5×10^{-4}	10 mL	30	Plasma	3
ZIF-8 ferrocene	Fluorescence	2.3×10^{6}	2 mL	25	Fortified ACSF	4
	Electrochemical	226				
$[Ru(bpy)_3]^{2+}$-UiO-66 and MIL-101 MOFs	Electrochemiluminescence	3.3×10^{-3}	8 µL	120	Fortified serum	5
g-C_3N_4 and Pd nanoparticles coated MIL-53-NH_2 MOFs	Electrochemiluminescence	3.3×10^{-3}	6 µL	45	Serum	6
Thioflavine T@Er-MOF	Fluorescence	641	-	-	Fortified serum	7
Luminol-Terbium-guanine monophosphate-Cu composite	Fluorescence	903	40 µL	30	Fortified serum	8
Quinine@ZIF-8@Fe_3O_4 magnetic propelled microswimmers	Fluorescence	140	10 µL[a] – 1 µL[b]	10	CSF	This work

[a]Volume for incubation. [b]Volume by measure.

Table S2. Demographic and clinical data of the participants.

Characteristics	Control (n=3)	MCI (n=4)	AD (n=6)	p-value
Age. Mean (SD), y	57.66 (2.88)	77 (4.54)	79.83 (1.33)	<0.0001
Female sex, n (%)	2 (66.66)	2 (50)	4 (57.14)	ns
Disease duration, mean (SD), y	-	1.5 (0.57)	3.16 (0.98)	<0.01
MMSE score, mean (SD)	29.66 (0.57)	26.25 (1.70)	19.00 (4.81)[a,b]	<0.01
CDR, mean (SD)	0	0.5	1.83 (0.75)	NA
CSF Aβ_{1-42}, pg/ml	840.97 (375.29)	546.66 (201.82)	317.52 (78.47)[c]	<0.05
CSF t-tau, pg/ml	230.49 (178.09)	834.28 (374.14)[c]	813.92 (409.88)[c]	<0.05

Abbreviations: MCI, mild cognitive impairment; AD, Alzheimer's disease dementia; SD, Standard Deviation; y, years; MMSE, Mini-Mental State Examination; CDR, Clinical Dementia Rating; ns, non-significant; NA, not applicable; t-tau, total tau.

[a]$p <$ 0.01 vs. Control

[b]$p < 0.05$ vs. MCI

[c]$p < 0.05$ vs. Control

Table S3. CSF sample analysis

	CSF samples	Aβ [pg/mL]		Tau [pg/mL]
		Microswimmers	ELISA	ELISA
C1	Control 69<br>Healthy, male, age: 56 years	616.9 ± 22.3	780.1	135.6
C2	Control 71<br>Healthy, female, age: 61 years	849.4 ± 31.9	1243.0	435.9
C3	Control 81<br>Healthy, female, age: 56 years	313.3 ± 19.9	499.8	120.0
P1	Patient 252<br>MCI, female, age: 83 years	707.9 ± 102.8	604.6	606.5
P2	Patient 262<br>MCI, female, age: 77 years	1012.7 ± 28.1	800.4	717.8
P3	Patient 326<br>MCI, male, age: 76 years	476.4 ± 13.9	445.2	622.3
P4	Patient 363<br>MCI, male, age: 72 years	342.7 ± 34.1	336.4	1390.6
P5	Patient 185<br>AD, male, age: 80 years	232.8 ± 26.3	313.5	482.9
P6	Patient 393<br>AD, female, age: 80 years	414.7 ± 15.1	399.3	1392.2
P7	Patient 187<br>AD, female, age: 82 years	118.4 ± 32.1	197.8	836.8
P8	Patient 197<br>AD, female, age: 78 years	260.2 ± 63.4	257.3	1043.1
P9	Patient 200<br>AD, male, age: 79 years	277.1 ± 19.5	389.8	233.9
P10	Patient 273-2<br>AD, female, age: 80 years	368.7 ± 19.7	347.4	894.5

(1) Han, J.; Zhang, M.; Chen, G.; Zhang, Y.; Wei, Q.; Zhuo, Y.; Xie, G.; Yuan, R.; Chen, S. Ferrocene Covalently Confined in porous MOF as Signal Tag for Highly Sensitive Electrochemical Immunoassay of amyloid-β. *J. Mater. Chem. B* **2017**, *5* (42), 8330-8336.
(2) Gordón Pidal, J. M.; Moreno-Guzmán, M.; Montero-Calle, A.; Valverde, A.; Pingarrón, J. M.; Campuzano, S.; Calero, M.; Barderas, R.; López, M. Á.; Escarpa, A. Micromotor-Based Electrochemical Immunoassays for Reliable Determination of Amyloid-β (1–42) in Alzheimer's Diagnosed Clinical Samples. *Biosens. Bioelectron.* **2024**, *249*, 115988.
(3) Supraja, P.; Tripathy, S.; Vanjari, S. R. K.; Singh, R.; Singh, V.; Singh, S. G. Label-Free Detection of β-Amyloid (1-42) in Plasma Using Electrospun SnO_2 Nanofiber Based Electro-Analytical Sensor. *Sens. Actuat. B. Chem.* **2021**, *346*, 130522.
(4) Qin, J.; Cho, M.; Lee, Y. Ferrocene-Encapsulated Zn Zeolitic Imidazole Framework (ZIF-8) for Optical and Electrochemical Sensing of Amyloid-β Oligomers and for the Early Diagnosis of Alzheimer's Disease. *ACS Appl. Mater. Interfaces* **2019**, *11* (12), 11743-11748.
(5) Dong, X.; Zhao, G.; Li, X.; Fang, J.; Miao, J.; Wei, Q.; Cao, W. Electrochemiluminescence Immunosensor of "Signal-Off" for β-amyloid Detection Based on Dual Metal-Organic Frameworks. *Talanta* **2020**, *208*, 120376.
(6) Fang, J.; Zhao, G.; Dong, X.; Li, X.; Miao, J.; Wei, Q.; Cao, W. Ultrasensitive electrochemiluminescence immunosensor for the detection of amyloid-β proteins based on resonance energy transfer between g-C_3N_4 and Pd NPs coated NH_2-MIL-53. *Biosens. Bioelectron* **2019**, *142*, 111517.
(7) Wang, X. Z.; Du, J.; Xiao, N. N.; Zhang, Y.; Fei, L.; LaCoste, J. D.; Huang, Z.; Wang, Q.; Wang, X. R.; Ding, B. Driving Force to Detect Alzheimer's Disease Biomarkers: Application of a Thioflavine T@Er-MOF Ratiometric Fluorescent Sensor for Smart Detection of Presenilin 1, Amyloid β-Protein and Acetylcholine. *Analyst* **2020**, *145* (13), 4646-4663.
(8) Liu, X.; Li, X.; Xu, S.; Guo, S.; Xue, Q.; Wang, H. Efficient Ratiometric Fluorescence Probe Based on Dual-Emission Luminescent Lanthanide Coordination Polymer for Amyloid β-Peptide Detection. *Sens. Actuat. B. Chem.* **2022**, *352*, 131052.
